# Antioxidant Peptides from *Sepia esculenta* Hydrolyzate Attenuate Oxidative Stress and Fat Accumulation in *Caenorhabditis elegans*

**DOI:** 10.3390/md18100490

**Published:** 2020-09-25

**Authors:** Xuesong Yu, Qina Su, Tianqi Shen, Qiong Chen, Ying Wang, Weizhang Jia

**Affiliations:** 1 School of Biosciences & Biopharmaceutics and Guangdong Province Key Laboratory for Biotehnology Drug Candidates, Guangdong Pharmaceutical University, Guangzhou 510006, China; axuesong@163.com (X.Y.); suqina123@126.com (Q.S.); shentianqiskylar@163.com (T.S.); 15699321850@163.com (Q.C.); 2 Institutes for Life Sciences and School of Medicine, South China University of Technology, Guangzhou 510641, China; wangying2@scut.edu.cn

**Keywords:** antioxidant peptides, oxidative stress, fat accumulation, *Sepia esculenta*, *Caenorhabditis elegans*

## Abstract

The hydrolysate of golden cuttlefish (*Sepia esculenta*) was prepared by using papain, and then, it was further separated by ultrafiltration, gel filtration chromatography, and reverse-phase high-performance liquid chromatography (RP-HPLC). The peptide components of the active fraction were identified by liquid chromatography-tandem mass spectrometry (LC-MS/MS), and then two novel peptides, SeP2 (DVEDLEAGLAK, 1159.27 Da) and SeP5 (EITSLAPSTM, 1049.22 Da), were obtained and displayed significant alleviation effects on oxidative stress in *Caenorhabditis elegans*. Studies indicated that *S. esculenta* antioxidant peptides (SePs) increase superoxide dismutase (SOD) activity but reduce reactive oxygen species (ROS) and malondialdehyde (MDA) levelsin oxidation-damaged nematodes. Using transgenic CF1553 nematodes, the *sod-3p*::GFP expression in the worms treated with SePs was significantly higher than that of the control nematodes. Real-time PCR also demonstrated that the expression of stress-related genes such as *sod-3* is up-regulated by SePs. Furthermore, studies showed that SePs could obviously decrease fat accumulation as well as reduce the elevated ROS and MDA levels in high-fat nematodes. Taken together, these results indicated that SePs are capable of the activation of antioxidant defense and the inhibition of free radicals and lipid peroxidation, play important roles in attenuating oxidative stress and fat accumulation in *C. elegans*, and might have the potential to be used in nutraceutical and functional foods.

## 1. Introduction

Reactive oxygen species (ROS) including hydrogen peroxide (H_2_O_2_), superoxide anions (O_2_-), and hydroxyl radical (OH) are produced inevitably in aerobic organisms, and subsequent processing via the antioxidant defense system is a highly complex and delicately balanced process [1,2]. The excessive ROS destroys the homeostasis with the cellular antioxidant defense system leading to oxidative stress, and the results of redox imbalance are often associated with damage to a wide range of macromolecules such as proteins, lipids, and nucleic acids [3,4]. The damaging effects of excessive ROS could be reduced by intracellular antioxidant enzymes including superoxide dismutases (SODs), glutathione peroxidases (GPXs), and catalases (CATs), as well as some non-enzymatic antioxidant molecules such as glutathione (GSH), vitamin C, and E [5,6]. However, subjected to severe stressors such as toxins and oxidants, the endogenous antioxidants may not be sufficient to counteract the destructive effects of ROS, and the intake of dietary antioxidants has been reported to prevent oxidative injury and maintain redox balance [7,8,9,10].

There is growing interest in the therapeutic applications of food-derived bioactive substances as safer alternatives, either as nutraceuticals or functional foods [11,12,13]. Bioactive peptides are a class of multifunctional compounds derived from food proteins, which have been reported to possess a variety of important physiologically related biological activities such as antioxidant, antihypertensive, and anti-inflammatory actions [14,15,16]. As a result, they have the potential to provide better alternatives to synthetic drugs to prevent and treat chronic diseases that affect a growing number people [15,17]. Marine products and by-products are important protein resources that can be used for the production of bioactive peptides using enzymatic hydrolysis [18,19]. Marine antioxidant peptides as free radical scavengers play significant roles in inhibiting lipid peroxidation, activating antioxidant enzyme, and maintaining redox balance [11,20]. Therefore, the preparation, purification, and structure–activity relationship of marine antioxidant peptides have attracted more and more attention, as they can be used as pharmaceuticals and nutraceuticals as well as active constituents for application in functional foods [21,22]. 

The golden cuttlefish (*Sepia esculenta*), as one of the most economically important cephalopod species in Japan, South Korea, and China, is rich in high-quality protein that can be used for the preparation of functional peptides by hydrolysis. Nevertheless, except for squid ink peptide (SHP, LKEENRRRRD) [23], few studies have been done on the hydrolysate of *S. esculenta*, and little information has been available on the peptide sequences in the hydrolysate. Therefore, the aim of this study is to prepare and separate peptides from the enzymatic hydrolysate of *S. esculenta*, and to research the antioxidant effects of bioactive peptides using *Caenorhabditis elegans*, which is a favorable model organism in the screening of various active compounds [24]. Studies indicated that *S. esculenta* antioxidant peptides could enhance antioxidant potential as well as attenuate oxidative stress and fat accumulation in *C. elegans*, and they have the potential to be used in nutraceutical and functional foods.

## 2. Results and Discussion

### 2.1. Antioxidant Activities of the Hydrolysate and Ultrafiltration Fractions

**Figure 1:**
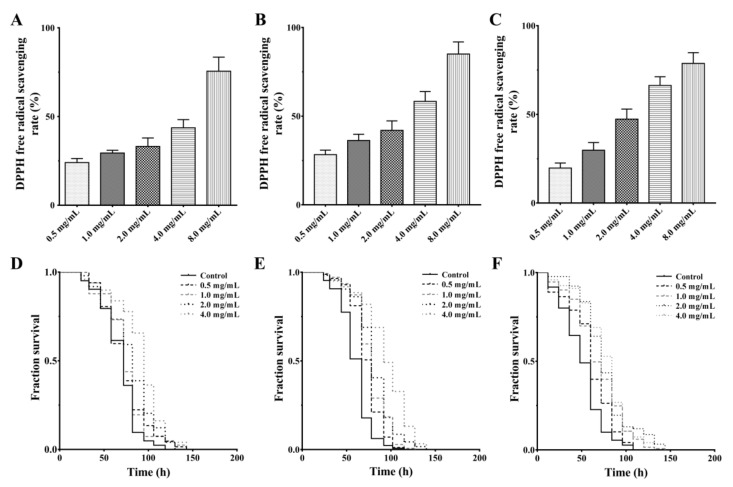
Antioxidant effects of the hydroysate and ultrafiltration fractions. (**A**) The 1,1-diphenyl-2-picrylhydrazyl (DPPH) free radical-scavenging effect of the enzymatic hydroysate. (**B**) The DPPH free radical-scavenging effect of the <3 kDa fraction. (**C**) The DPPH free radical-scavenging effect of the >3 kDa fraction. (**D**) The effect of the enzymatic hydrolysate on the survival of oxidation-damaged worms. (**E**) The effect of the <3 kDa fraction on the survival of oxidation-damaged worms. (**F**) The effect of the >3 kDa fraction on the survival of oxidation-damaged worms. The L4-stage wild-type worms were pretreated with or without samples at the specified concentration (0.5, 1.0, 2.0, and 4.0 mg/mL) for 24 h at 20 °C, and further treated with 50 mM paraquat. The survival was counted every 12 h until all worms were dead. The result is shown in a Kaplan–Meier curve, and the significance is compared by a log-rank test.

Peptides have gained significant interest in recent years due to their various beneficial effects on human health [14,25]. Enzymatic hydrolysis is an important approach for the production of functional peptides from a variety of food proteins [7,26]. In this study, S esculenta was hydrolyzed using papain for the preparation and separation of antioxidant peptides. As shown in Figure 1A, the hydrolysate has the capacity of scavenging 1,1-diphenyl-2-picrylhydrazyl (DPPH) free radical in a dose-dependent manner at concentrations ranging from 0.5 to 8 mg/mL (EC50 3.52 mg/mL). Studies have shown that a low molecular weight (MW) fraction has more potent antioxidant activity [11]. Therefore, the <3 kDa and >3 kDa ultrafiltration fractions were recovered through an ultrafiltration membrane with the molecular weight cut off (MWCO) of 3 kDa. Activity analysis showed that <3 kDa enzymatic hydrolysate has a higher DPPH free radical-scavenging activity than that of the >3 kDa fraction (Figure 1B,C, EC50 2.09 and 2.17 mg/mL, respectively). Although the DPPH radical scavenging test cannot fully reflect the in vitro antioxidant activity of the enzymatic hydrolysate, it suggests that the hydrolysate can be used as a free radical scavenger to inhibit the DPPH chain reaction [22]. Moreover, these results are supported by previous studies; that is, low MW enzymatic hydrolysate easily reacts with free radicals and terminates the free radical chain reaction [27].

It is common practice to use a model organism in the assessment of the anti-oxidative stress capacity of compounds [8]. Therefore, an oxidation-damaged model of *C. elegans* induced by paraquat was constructed to detect the antioxidant activity of the hydrolysate. When pretreated with *S. esculenta* hydrolysate, the survival of worms increase significantly compared to that of the control group with a dose-dependent effect (Figure 1D), suggesting that the hydrolysate confers a protective effect against oxidative stress in worms. To obtain oxidative stress-resistant fractions, the *S. esculenta* hydrolysate was isolated into <3 kDa and >3 kDa enzymatic fractions, and both fractions were tested for their anti-oxidative stress capacity through the paraquat resistance assay. As shown in Figure 1E,F, treatment with either <3 kDa or >3 kDa enzymatic hydrolysate can increase the survival rate of worms, and the part of <3 kDa is slightly better. It has been reported that antioxidant enzymes are thought to protect the cell from the deleterious effects of various radicals. Therefore, the hydrolysate and peptide fractions attenuate 1,1′-dimethyl-4,4′-bipyridinium dichloride (PQ)-induced radicals toxicity, which may be related to the activation of antioxidant defense [20,22]. Together, these results indicate that the <3 kDa enzymatic hydrolysate is a major fraction that is potentially responsible for the antioxidant activity of the hydrolysate.

### 2.2. Antioxidant Activities of Peptide Fractions Obtained by Gel Filtration Chromatography

**Figure 2:**
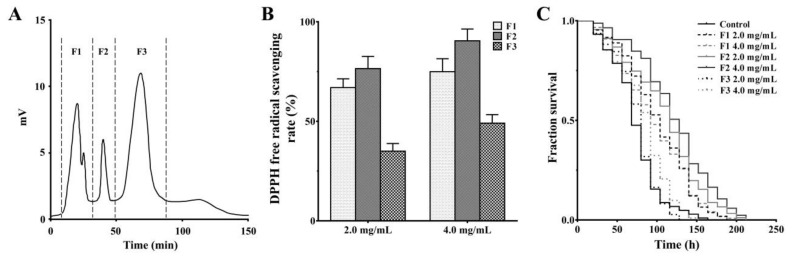
Antioxidant effects of peptide fractions purified by gel filtration chromatography. (**A**) The elution profile of the <3 kDa fraction by gel filtration on Sephadex G-25. (**B**) The DPPH free radical-scavenging effects of peptide fractions F1, F2, and F3. (**C**) The effects of peptide fractions F1, F2, and F3 on the survival of oxidation-damaged worms. As stress assay in Figure 1, the L4-stage wild-type worms were cultured with or without peptide samples (2 and 4 mg/mL, respectively) for 24 h, and further treated with paraquat. The result is shown in a Kaplan–Meier curve, and the significance is compared by a log-rank test.

Gel filtration chromatography is a popular technique that separates peptides based on the molecular weight (MW) of each component [7,9,28]. As shown in Figure 2A, the <3 kDa fraction with the higher antioxidant activity was subsequently purified into three subfractions (F1, F2, and F3) using a column packed with Sephadex G-25. These fractions were pooled, freeze-dried, and analyzed for their DPPH free radical-scavenging activity, and the oxidative stress resistance in oxidation-damaged worms. Compared with fractions F1 and F3 (Figure 2B), fraction F2 exhibits a significantly higher DPPH free radical-scavenging activity (EC500.846 mg/mL,). At the same time, treatments with 2 and 4 mg/mL of the fractions F1, F2, and F3 has the ability to increase the survival rate of the oxidation-damaged worms, especially fraction F2 with better anti-oxidative stress activity (Figure 2C). It has been reported that peptides with low MW can interact with free radicals more effectively in the oxidation process [29,30]. However, after further separation of <3 kDa fraction by Sephadex-G25 gel filtration chromatography, the antioxidant activity of the peptide is closely related to the main active components in the peptide mixture, but it is not completely dependent on the MW distribution [31]. Fraction F2 exhibits significant DPPH free radical-scavenging activity and the capacity to alleviate oxidative stress. Therefore, the peptides in this most active fraction were further separated by reverse-phase high performance liquid chromatography (RP-HPLC).

### 2.3. Antioxidant Activities of Peptide Fractions Obtained by RP-HPLC

**Figure 3:**
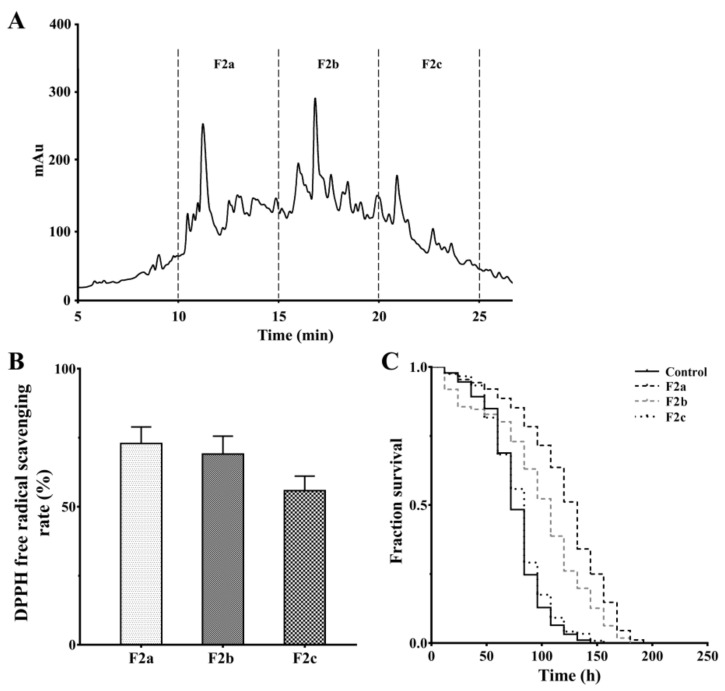
Antioxidant effects of peptide fractions purified by reverse-phase high performance liquid chromatography (RP-HPLC). (**A**) RP-HPLC chromatography profile of fraction F2. (**B**) The DPPH free radical-scavenging effects of subfractions F2a, F2b, and F2c (2 mg/mL, respectively). (**C**) The effects of subfractions F2a, F2b, and F2c from fraction F2 on the survival of oxidation-damaged worms. As the stress assay in Figure 1, the L4-stage wild-type worms were cultured with or without peptide samples (2 mg/mL, respectively) and further treated with paraquat. The result is shown in a Kaplan–Meier curve, and the significance is compared by a log-rank test.

Reversed-phase high-performance liquid chromatography (RP-HPLC) has been proven to be extremely versatile for the isolation and purification of peptides [9,11]. Therefore, through the ultrafiltration and gel filtration chromatography, the potential fraction F2 was separated by RP-HPLC on an analytical C18 column. The profile of F2 is presented in Figure 3A. According to the elution time, 3 peptide fractions (F2a, F2b, and F2c) were collected separately and freeze-dried to detect their antioxidant effects. As shown in Figure 3B, it is noteworthy that fraction F2a has a higher DPPH free radical-scavenging activity than those of fractions F2b and F2c (2 mg/mL, respectively). At the same time, fraction F2a has significant anti-oxidative stress activity and is better than other peptide fractions at 2 mg/mL (Figure 3C). To sum up, the F2a was subjected to LC-MS/MS analysis for peptide identification.

### 2.4. Identification, Synthesis, and Antioxidant Activities of Peptides

**Figure 4:**
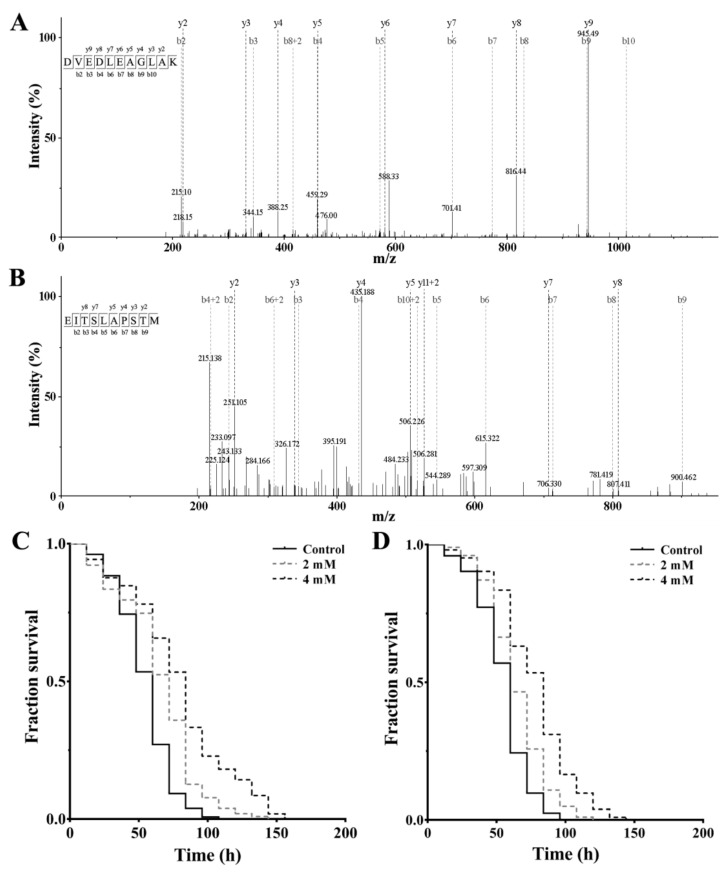
MS/MS spectra and anti-oxidative stress activities of peptide SeP2 and SeP5. (**A**) MS/MS spectra of peptide SeP2. The MS/MS spectra at *m*/*z* 580.30 corresponds to the [M + 2H]^2+^ ion of peptide SeP2 (DVEDLEAGLAK). (**B**) MS/MS spectra of peptide SeP5. The MS/MS spectra at *m*/*z* 525.26 corresponds to the [M + 2H]^2+^ ion of peptide SeP5 (EITSLAPSTM). (**C**) The effect of peptide SeP2 on the survival of oxidation-damaged worms. (**D**) The effect of peptide SeP5 on the survival of oxidation-damaged worms. As the stress assay in Figure 1, the L4-stage wild-type worms were cultured with or without peptide samples (2 and 4 mM, respectively) and further treated with paraquat. The result is shown in a Kaplan–Meier curve, and the significance is compared by a log-rank test.

In order to obtain the peptide sequences, the peptide fraction F2a was analyzed by RP-nano-LC-MS/MS, which has become an important tool for the identification of peptide mixtures [11,20]. As presented in Table 1, 8 peptides were obtained through database-assisted peptide sequencing, which is consistent with previous studies—that is, the peptide composition is less than 20 amino acid (aa) residues [7]. For instance, the MS/MS spectrum of the SeP2 (DVEDLEAGLAK, 1159.27 kDa) and SeP5 (EITSLAPSTM, 1049.22 kDa) were shown in Figure 4A,B, respectively. The observed mass values are consistent with the calculated values within 1.0 mass unit, SeP2: MW_calcd_ 1159.27, *m*/*z*_calcd_ [M + 2H]^2+^ 579.64, *m*/*z*_obsd_ [M + 2H]^2+^ 580.3; SeP5: MW_calcd_ 1049.22, *m*/*z*_calcd_ [M + 2H]^2+^ 1159.27, *m*/*z*_calcd_ [M + 2H]^2+^ 579.64, *m*/*z*_obsd_ [M + 2H]^2+^ 580.3; SeP5: MW_calcd_ 1049.22, *m*/*z*_calcd_ [M + 2H]^2+^ 524.61, *m*/*z*_obsd_ [M + 2H]^2+^ 525.26. Most of the C-terminus of the identified peptide are R and K, but the C-terminus of the identified SeP5 and SeP6 are M and E, which may be due to the wide specificity of papain [32]. It is interesting that 7 peptides identified in this fraction originate from a myosin heavy chain; this is also seen in the study performed by Xing et al. 2018, where the highest number of peptides identified in ham hydrolysate showing high antioxidant activity was from myosin protein [33].

In an attempt to test the peptides potentially responsible for the observed antioxidant activity in the active fraction F2a, the obtained peptides were synthesized and screened for their anti-oxidative stress activities at the indicated concentration of 2 and 4 mM, respectively. As shown in Figure 4C,D, SeP2 and SeP5 have the ability to increase the survival of oxidation-damaged worms in a dose-dependent manner. At present, the structure–activity relationship of antioxidant peptides has not yet been fully elucidated. However, the presence of hydrophobic aa residues including Val, Leu, Ile, Pro, Ala, Met, and Gly within a peptide is believed to influence the antioxidant activity [34,35]. Therefore, hydrophobic aa residues in peptides DVEDLEAGLAK (Val, Leu, Ala and Gly) and EITSLAPSTM (Ile, Leu, Ala, Pro and Met) play a crucial for their antioxidant activities. In addition, it has been reported that the polar aa residues such as Glu, Asp, and Lys play a key role in the OH scavenging of antioxidant peptides [9]. So, the Glu, Asp, and Lys residues in DVEDLEAGLAK and Glu residue in EITSLAPSTM may contribute to the elimination of hydroxyl radicals. Meanwhile, the position of aa residue is also pivotal in peptide antioxidant activity [36]. Therefore, it is possible that the antioxidant activity observed with SeP2 and SeP5 may be associated with the presence hydrophobic aa residues within their sequences. It is suppose that the bulky hydrophobic aa residues (Ala-Gly-Leu-Ala) at the C-terminal region of SeP2 is believed to contribute to the anti-oxidative stress activity. For SeP5, it is possible that the bulky hydrophobic aa residues such as Leu-Ala-Pro in the middle part should play a critical role in its anti-oxidative stress capacity.

### 2.5. Increase of SOD Activity and Decrease of ROS and MDA Levels in Oxidation-Damage Nematodes

**Figure 5:**
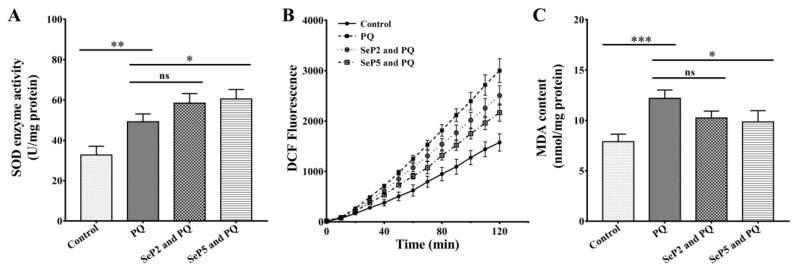
Increase of superoxide dismutase (SOD) activity and decrease of reactive oxygen species (ROS) and malondialdehyde (MDA) levels in oxidation-damage worms. (**A**) The effects of SePs on SOD activities. (**B**) The effects of SePs on ROS levels. (**C**) The effects of *S. esculenta* antioxidant peptides (SePs) on MDA levels. The L4-stage wild-type worms were cultured with or without SePs (4 mM, respcectively) at 20 °C for 24 h and then treated with 10 mM paraquat. The worms were collected and washed with M9 and then homogenized in PBST buffer. The worm lysate was used for the detection of SOD activity and MDA levels. The results are expressed as mean ± SEM from three repeated experiments, and the statistical significance calculated by one-way ANOVA. *: *p* < 0.05; **: *p* < 0.01; ***: *p* < 0.001.

Aerobic organisms have a number of antioxidant defenses to minimize the damaging effects of excessive ROS, in which SOD is known to be a major ROS scavenging enzyme protecting cells from free radical-induced damage [1,37]. Therefore, the effects of *S. esculenta* antioxidant peptides (SePs) on SOD activities were detected in oxidation-damaged worms. As shown in Figure 5A, compared with the paraquat-treated group, the SOD activity was increased significantly when the worms were co-treated with paraquat and SePs. These results suggest that SePs show antioxidant activities by modulating intracellular antioxidant defense. Some specific aa residues in peptides, such as hydrophobic aa, are considered to play a key role in enhancing the activity of antioxidant enzymes. In the future, the effects of these aa residues can be verified by the substitution of aa to further determine their roles in antioxidant activity. Since paraquat causes oxidative stress via increasing ROS generation [38], we then tested whether the alleviation of oxidative stress of SePs in oxidation-damaged worms were through decreasing the ROS level. The wild-type worms were treated with paraquat and SePs, and the ROS levels were determined by the DCFH-DA method. As shown in Figure 5B, when the worms were co-treated with paraquat and SePs, the increase of the ROS level in the paraquat group was significantly reduced, indicating that the SePs were capable of decreasing the elevated ROS level induced by paraquat. It is worth noting that natural antioxidant compounds such as C-phycocyanin and quercetin can also be used in the treatment against paraquat-induced damage [38,39]. The main mechanisms involved in the protective effects of these antioxidants include the reduction of oxidative stress and induction of antioxidant defenses. Therefore, our results suggest that the antioxidant peptides identified in our study, as well as other antioxidant peptides reported elsewhere [34,40,41], show better antioxidant activity through the reinforcement of antioxidant defense and the reduction of deleterious ROS production. At the same time, malonaldehyde (MDA), which is considered to be an important bio-marker for oxidative stress, was characterized with a certain degree of decline (Figure 5C). Overall, SePs could reduce the oxidative stress injury by the activation of antioxidant enzymes, inhibition of free radicals, and lipid peroxidation [42,43].

### 2.6. Regulation of Stress-Related Genes Expression

**Figure 6:**
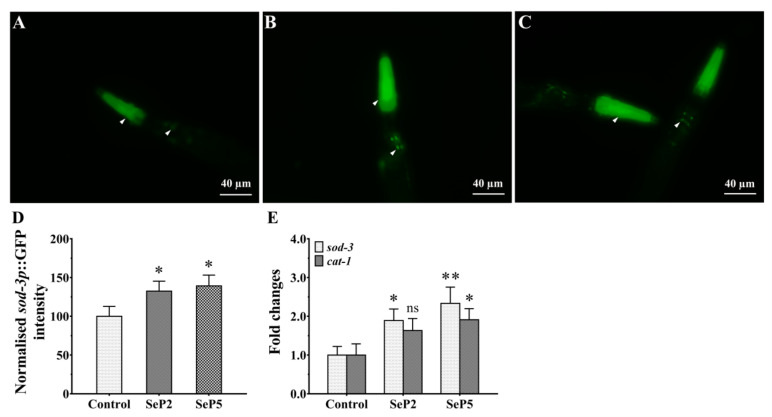
Measurement of stress-associated gene expression by SePs. (**A**–**C**) Representative images of the *sod-3p*::GFP expression in transgenic CF1553 worms. The L1-stage transgenic CF1553 worms were cultured in S. medium containing NA22 at 20 °C for 24 h and further treated with SePs for 24 h. The worms were anesthetized in M9 containing sodium azide (1%), and then the fluorescence images were detected. (**D**) Quantified *sod-3p*::GFP fluorescence intensity. The collected worms were washed with M9 and homogenized in PBST buffer. Fluorescence intensity is determined by a Fluoroskan Ascent FL plate reader at an excitation of 485 nm and emission of 530 nm. (**E**) The effects of SeP2 and SeP5 on the gene expressions of sod-3 and cat-1 in N2 worms. The L1-stage wild-type worms were cultured for 24 h at 20 °C and further cultured with or without SePs (4 mM, respectively). The results are expressed as mean ± SEM from three repeated experiments, and the statistical significance is calculated by one-way ANOVA. Scale bar = 40 µm. *: *p* < 0.05, **: *p* < 0.01.

Since *S. esculenta* antioxidant peptides were shown as above to enhance oxidative stress resistance by increasing SOD activity and modulating ROS detoxification, we used the transgenic nematodes CF1553 that express a *sod-3p*::GFP reporter to determine the effects of peptides on SOD-3 expression. As shown in Figure 6D, the relative fluorescence intensity of the transgenic nematodes CF1553 treated with SePs were both significantly higher than that of the control worms, indicating that SePs can effectively induce the expression of antioxidant enzymes. In order to further confirm antioxidant properties, we investigated the effects of SePs on the transcriptions of stress-related genes sod-3 and cat-1. As shown in Figure 6E, the results showed that sod-3 was up-regulated by SeP2 and SeP5, while ctl-1 was also up-regulated by SeP5. It has been reported that some antioxidants could increase the oxidative stress resistance through the activation of antioxidant related signaling pathways and sequentially up-regulate the expressions of their target genes such as sod-3 and cat-1, which might enhance the potential of anti-oxidative stress [44,45,46]. In agreement with these, the present results demonstrate that *S. esculenta* antioxidant peptides have a protective effect in oxidation-damaged worms, which is associated with the up-regulation expression of stress-related genes, suggesting that peptides alleviating oxidative stress are closely related to enhancing antioxidant activity in *C. elegans*.

### 2.7. Decreased Fat Accumulation, ROS, and MDA Levels in High-Fat Nematodes

**Figure 7:**
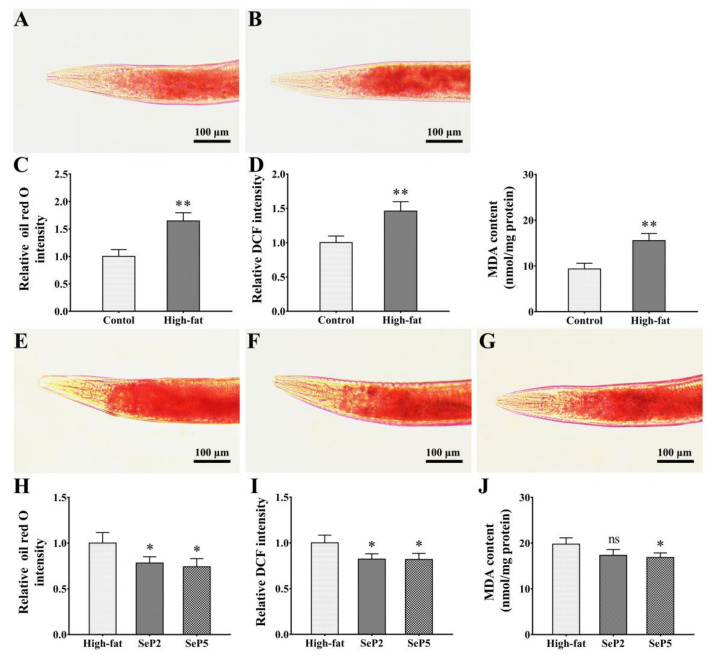
Effects of SePs on fat accumulation, ROS, and MDA levels in high-fat worms. (**A**,**B**) Representative images of fat accumulation in normal and high-fat worms by Oil Red O staining. (**C**) Relative fat quantification of normal diet and high-fat worms by Oil Red O staining. (**D**) The ROS levels of normal diet and high-fat worms. (**E**) The MDA levels of normal diet and high-fat worms. The wild-type worms were induced with or without glucose (10 mM) on NGM plates containing *E. coli* OP50 strain for 3 days from the L1 stage. (**F**–**H**) Representative images of fat accumulation after treatment with or without SePs in high-fat worms by Oil Red O staining. (**I**) Relative fat quantification in high-fat worms treated with or without SePs. (**J**) The ROS level in high-fat worms treated with or without SePs. (**K**) The MDA level in high-fat worms treated with or without SePs. The L1-stage worms were cultured on NGM plates containing OP50 and 10 mM glucose until the L4 stage; then, the worms were transferred to 24-well plates and cultured in S. medium containing *E. coli* NA22, 5-fluoro-2-deoxyuridine (5-FUDR) (75 µg/mL), glucose (10 mM), and SePs (4 mM, respectively), and further cultured for 3 days. The images were quantified using ImageJ software, and the assay was repeated three times; more than 20 worms were measured per assay. The results are expressed as mean ± SEM from three repeated experiments, and the statistical significance was calculated by unpaired *t* test or one-way ANOVA. *: *p* < 0.05; **: *p* < 0.01.

Lipid droplets are stored in the intestinal and hypodermal cells in *C. elegans*, which can be clearly visualized after staining by Oil Red O [47]. The high-fat *C. elegans* models constructed by the administration of 10 mM glucose were used to measure fat accumulation by Oil Red O staining [48]. As shown in Figure 7C, the worms treated with glucose exhibited a significant increase in the content of body fat, which suggested that the high-fat *C. elegans* model was established successfully. At the same time, the ROS and MDA levels were significantly increased in high-fat worms compared to that in normal worms (Figure 7D,E). Overall, excessive glucose intake disturbs the energy homeostasis in *C. elegans*.

It has been demonstrated that the accumulation of fat promotes the generation of ROS, and increased ROS production contributes to an excessive accumulation of fat [49]. Considering the anti-oxidative stress capacity, the effects of SePs on fat accumulation, ROS, and MDA levels in high-fat worms was examined. When the worms were treated with SePs, the increase of fat accumulation was significantly reduced, indicating that the supplementation of SePs was capable of decreasing the elevated lipid level induced by glucose (Figure 7I). Moreover, as shown in Figure 7J,K, the ROS and MDA levels were also decreased in different degrees by SePs treatment, suggesting that SePs could enhance the ability of ROS scavenging and lessen lipid peroxidation in high-fat worms. It has been reported that hydrolysate could improve glucose homeostasis in a mouse model of high-fat diet-induced obesity [43]. In this study, it can be inferred that the fat accumulation reduction by SePs could be related to its antioxidant activities and the scavenging ability of ROS. Overall, *S. esculenta* antioxidant peptides have the function of inhibiting excessive fat accumulation, eliminating free radicals, and decreasing lipid peroxidation end products such as MDA; thus, they may have beneficial potentials in promoting health.

The importance of nutrition and the benefits of dietary supplements to health promotion and disease prevention have been documented increasingly in specific studies [14,50]. Many studies have shown that antioxidant peptides could effectively enhance the oxidative stress resistance of organisms by neutralizing free radicals, prohibiting oxidative chain reactions, and regulating multiple stress-responsive pathways and gene expressions [51,52,53]. Combined with the results of the present studies, peptides as an important class of antioxidants are capable of providing protective effects against oxidative stress and fat accumulation, which have the potential to be used in nutraceutical and functional foods for disease prevention.

## 3. Materials and Methods

### 3.1. Materials and Chemical Regents

The golden cuttlefish (*Sepia esculenta*) was bought from Huangsha Aquatic Market Co. (Guangzhou, China). After removing viscera and bones, the cuttlefish sample was cut into small pieces, mixed with deionized water, and then homogenized at 4 °C (15 s three times at 5 s intervals). The homogenate material was lyophilized and placed at −80 °C until it was used for enzymatic hydrolysis.

Papain in this work was bought from Shanghai Aladdin Biochemical Technology Co., Ltd. (China). A 3 kDa cutoff ultracentrifuge tube was obtained from Millipore (Bedford, MA, USA). Sephadex G25 medium for gel filtration chromatography was purchased from GE Healthcare Bio-Sciences AB (Uppsala, Sweden). Trifluoroacetic acid (TFA) was obtained from Beijing Solarbio Science and Technology Co. Ltd. (Beijing, China). Acetonitrile used for liquid chromatography was obtained from Merk Co. (Darmstadt, Germany). The 5-FUDR (5-fluoro-2-deoxyuridine), paraquat (1,1′-dimethyl-4,4′-bipyridinium dichloride, PQ) and DPPH (1,1-diphenyl-2-picrylhydrazyl) were obtained from Sigma-Aldrich Chemical Co. (St. Louis, MO, USA). The TRIzol for total RNA extraction was obtained from Invitrogen Life Technologies (Carlsbad, CA, USA). The assay kits of PrimeScript™ RT reagent kit (RR047A) and SYBR Premix Ex Taq (DRR041A) were obtained from Dalian Takara Biotechnology Co. Ltd. (China). The assay kits for ROS level, SOD activity, MDA content, and protein concentration (bicinchoninic acid, BCA) were obtained from Shanghai Beyotime Biotechnology Co., Ltd. (China). Other chemicals and reagents used in this research were obtained from Guangzhou Chemical Reagent Co. (China), which are of analytical grade unless stated specially.

### 3.2. Strains and Maintenance

The *C. elegans* used in this work contain the following: wild-type strain N2 and transgenic strain CF1553. The study protocol was approved by the institutional ethics committee on animal research. *Escherichia coli* OP50 and NA22 were commonly used as a food source for worm growth. All *C. elegans* and *E. coli* strains were bought from the Caenorhabditis Genetics Center (University of Minnesota, MN, USA). The worms were cultured on an agar plate of *E. coli* OP50 containing cholesterol (5 µg/mL) and maintained in 15 °C or 20 °C incubator. The L1-stage synchronized worms were obtained through the alkaline hypochlorite method and cultured in M9 medium shaking all night. The high-fat worms were constructed as described with minor modification [48]. In short, the high-fat worms were induced on NGM solid plates containing *E. coli* OP50 strain and glucose (10 mM) for 3 days from the L1 stage; then, the worms were used to detect fat accumulation. For samples treatment, the L1-stage worms were cultured on NGM solid plates including OP50 and glucose (10 mM) until the L4 stage; then, the worms were transferred to 24-well plates and cultured in S. medium including *E. coli* NA22, 5-FUDR (75 µg/mL), glucose (10 mM), and peptide samples (4 mM), and further cultured for 3 days before a fat accumulation test.

### 3.3. Preparation of the Protein Hydrolysate of Sepia Esculenta

The lyophilized homogenate was redissolved in water (50 mg/mL) and further hydrolyzed with papain (3000 U/g). The conditions of papain enzymatic hydrolysis were slightly modified as follows: pH 7.0, hydrolyzed for 4 h at 55 °C [20]. After that, the hydrolysate was boiled for about 10 min to inactivate papain protease. The protein hydrolysate was placed at 4 °C overnight. The supernatant liquid was collected by suction filtration and centrifuged at 6000× *g* at 4 °C for 30 min. The final enzymatic hydrolytic solution is lyophilized and placed at −20 °C until for further experiments.

### 3.4. DPPH Radical-Scavenging Activity

The scavenging activity of DPPH free radical was determined by a slightly modified method [32]. In short, 0.1 mM DPPH solution is prepared in absolute ethanol. In a 96-well microplate, 0.1 mL DPPH solution was added to a sample with a series of concentrations (0.5, 1.0, 2.0, 4.0, and 8.0 mg/mL) for reaction. The blank group was treated with ethanol instead of DPPH solution. Furthermore, the control group contains DPPH but no samples. After 30 min of reaction at room temperature in the dark, the discoloration was measured at 517 nm. The activity of free radical scavenging (%) is calculated as follows:

Scavenging activity (%) = (A_control_ − A_sample_)/(A_control_ − A_blank_) × 100.

The A_control_, A_blank_, and A_sample_ are the absorbance of the control group, the blank group, and the test group of the specific concentration of samples.

### 3.5. Paraquat Survival Assay

A paraquat survival test using a *C. elegans* model was carried out as previously described [54]. The wild-type worms at the L1 stage were cultured to the L4 stage in S medium at 20 °C, and then 5-FUDR (75 µg/mL) was added. Approximately 20 worms were deposited in each well of 96-well microplates (approximately 100 worms for each group), which also contain *E. coli* NA22 as food and samples with or without a series of concentrations (0.5, 1.0, 2.0, and 4.0 mg/mL, respectively). After an additional 24 h of incubation, they were treated with 50 mM of paraquat. The number of live and dead worms were counted every 12 h until all of them died.

### 3.6. Separation of Antioxidant Peptide Fractions

The lyophilized hydrolysate was redissolved (20 mg/mL) and further separated into the >3 kDa and <3 kDa fractions through ultrafiltration tubes with 3 kDa MWCO. The <3 kDa fraction (10 mg/mL) has better antioxidant activities and was purified on a Sephadex G-25 gel chromatography column. Three fractions (F1, F2, and F3) were obtained and lyophilized for free radical-scavenging and oxidative stress assays at 2 and 4 mg/mL, respectively. The fraction F2 with better activity was separated by RP-HPLC on an Elite C18 column (5 µm, 4.6 × 250 mm, Elite Co. Ltd., Dalian, China) with a gradient of 5–30% acetonitrile containing trifluoroacetic acid (0.1%, *v*/*v*). Three subfractions (F2a, F2b and F2c) were obtained from the F2 fraction and freeze-dried for further activity measurement.

### 3.7. Identification of Peptide Sequences by LC-MS/MS

Reversed-phase nanocolumn liquid chromatography-tandem mass spectrometry (RP-nano-LC-MS/MS) was used to identify the peptide components in F2a, which presents better antioxidant activity. In short, the peptide sample was dissolved in solvent A (98:2 water/acetonitrile (*v*/*v*) containing 0.1% (v) formic acid) and loaded on a C18 NanoLC trap column (3 µm, 120 Å). Elution was performed with a gradient of 5–80% solvent B (2:98 water/acetonitrile (*v*/*v*) containing 0.1% (v) formic acid). LC-MS/MS detection was performed on a Triple TOF 5600 System (SCIEX, Concord, ON, Canada). The data file was searched collectively in the translated protein database of *S. esculenta* through the Protein Pilot Software v. 4.5. A confidence threshold of more than 95% and a false discovery rate (FDR) of less than 1% were selected for peptide identification.

### 3.8. Peptide Synthesis and Antioxidant Effects

The above-identified peptides were synthesized through solid-phase peptide synthesis (Top-Peptides Co. Ltd., Shanghai, China). The paraquat survival test was used to study the antioxidant activity of synthetic peptides at indicated concentrations (2 and 4 mM). The experiment refers to Section 3.5.

### 3.9. Detection of SOD Activity

L1-stage wild-type worms were cultured to the L4 stage in S medium inoculated with NA22 as food, and then 5-FUDR (75 µg/mL) was added. After that, the worms were cultured with or without SePs (4 mM) at 20 °C for 24 h and then further treated with paraquat (10 mM) for 24 h. The worms (approximately 2000) were collected, washed with M9, and then homogenized using a glass homogenizer in PBST (PBS containing 0.1% Tween 20). The supernatant was collected by centrifugation at 10,000 *g* for 5 min, and then used for the measurement of SOD activity by Beyotime Biotechnology test kits (Shanghai, China). A BCA protein test kit (Beyotime, China) was used to determine protein concentration, and the test results were normalized by protein content.

### 3.10. Measurement of ROS and MDA Levels

The ROS level was determined using the DCFH-DA as previously described [55]. In oxidation-damage assay, the worms were performed as described in Section 3.10. For fat accumulation assay, the worms were prepared using the process as described in Section 3.2. The worms (approximately 2000) were collected, washed with M9, and then homogenized using a glass homogenizer in PBST (PBS containing 0.1% Tween 20). The supernatant was collected by centrifugation at 10,000 *g* for 5 min, and the ROS level was measured using a commercial chemical analysis kit (Beyotime, China). After transferring 50 µL of worm lysate to a 96-well black microplate and incubated with 50 µL of DCFH-DA (100 µM), the fluorescence intensity (485 nm excitation and 535 nm emission, respectively) was measured on a Fluoroskan Ascent FL microplate reader (MA, USA). As for the determination of MDA content, L4-stage worms were cultured with or without SePs at 20 °C for 24 h and then treated with paraquat (10 mM) for 3 days. The MDA content was measured using a Lipid Peroxidation MDA Assay Kit (Beyotime, China). Protein concentration was detected with a BCA protein test kit, and the results were normalized by protein content.

### 3.11. Determination sod-3p::GFP Expression

As previously described [37], the expression level of SOD-3 was detected using the transgenic worm CF1553 expressing *sod-3p*::GFP reporter. At 20 °C, the L1-stage worms were treated with or without SePs until the L4 stage. In order to visually observe the expression of *sod-3p*::GFP, the worms were treated with sodium azide (1%), and the fluorescence images were obtained by the Zeiss Axio observer of ZEN software (Göttingen,, Germany). To quantify the expression level of *sod-3p*::GFP, worms were collected, washed with M9, and then homogenized with a glass homogenizer in PBS with 0.1% Tween 20 (*v*/*v*). The lysate was collected at 10,000× *g*, and the protein content was measured with the above-mentioned BCA protein test kit. The fluorescence intensity was detected with a Fluoroskan Ascent FL plate reader at an excitation of 485 nm and an emission of 535 nm.

### 3.12. Real-Time Quantitative PCR

The L1-stage wild-type worms were cultured in S. medium containing NA22 as food at 20 °C for 24 h and further cultured with SePs for 24 h. The worms were collected by centrifugation and then washed three times with M9. According to the manufacturer’s instructions, total RNA was extracted from the worms with TRIzol reagent, and the reverse transcription was performed with the PrimeScript™ RT reagent Kit. The real-time quantitative PCR was performed with SYBR Premix Ex Taq II Reagent Kit (TaKaRa) in a StepOne Plus Real-Time PCR Detection System (Carlsbad, CA, USA). The following gene specific primers were used for detection, *sod-3*, F: 5′-GAGCTGATGGACACTATTAAGCG-3′ and R: 5′-GCACAGGTGGCGATCTTCAAG-3′; *ctl-1*, F: 5′-TCTACTCGGATCGTGGAATTCCT-3′ and R: 5′-TTGGAACCTTGAGCAGGCTTG-3′; *β-actin*, F: 5′-CCACGAGACTTCTTACAACTCCATC-3′ and R: 5′-CTTCATGGTTGATGGGGCAAGAG -3′.

### 3.13. Oil Red O Staining in C. elegans

The Oil Red O staining was carried out according to the previous method with some modifications [48]. The worms in Oil Red O staining were prepared using the process as above described in Section 3.2. The worms were collected and washed three times using PBS buffer and treated with sodium azide solution (1%) for 10 min. Four percent hypoformaldehyde was used to fix at 4 °C for 15 min. The worms were then subjected to two freeze–thaw cycles, freezing for 20 min at −80 °C and thawing with water for 5 min. The paraformaldehyde was removed with PBS buffer and dehydrated with 60% isopropanol at room temperature for 15 min. After removing isopropanol, the worms were stained with Oil Red O staining solution [60% Oil Red O stock solution (0.5% of Oil Red O in isopropanol): 40% filterer water] at room temperature for 6 h. Stained worms were collected and washed twice for removing the dyes. The ImageJ software was used for quantitative analysis, and the result was displayed as the fat intensity of the stained worms. The test was repeated three times, and more than 20 worms were detected in each test.

### 3.14. Statistical Analysis

GraphPad Prism 7.0 version statistics software (GraphPad, San Diego, CA, USA) was used to perform analyses. The survival rate of the worms was analyzed by the Kaplan–Meier method and log-rank test. Quantitative data from gene expression are normalized by transcription levels of the internal reference (*β-actin*). Each assay was performed at least three times, and the statistical significance calculated by unpaired *t*-test or one-way ANOVA. A probability value of less than 0.05 was regarded as statistically significant.

## 4. Conclusions

In the present work, by ultrafiltration, gel filtration, RP-HPLC, and LC-MS/MS, two novel antioxidant peptides (SeP2 and SeP5) were isolated and identified from the enzymatic hydrolysate of *S. esculenta*, which display alleviation effects on paraquat-exposed oxidative stress in *C. elegans*. We found that *S. esculenta* antioxidant peptides were capable of increasing SOD activity and reducing the ROS level and MDA content in oxidation-damaged worms. Further experiments showed that SePs can increase the fluorescence intensity of the *sod-3p*::GFP reporter in transgenic CF1553 strain and induce the up-regulation expression of stress-related genes such as *sod-3* in wild-type worms. Furthermore, SePs can obviously decrease fat accumulation and reduce the elevated ROS and MDA levels in high-fat worms. Taken together, these findings demonstrated that SePs are capable of activating antioxidant defense and inhibiting free radicals and lipid peroxidation, play important roles in attenuating oxidative stress and fat accumulation in *C. elegans*, and have potential application prospects in nutraceutical and functional foods.

## Figures and Tables

**Table 1:** HPLC-MS/MS identification and solid-phase synthesis of *S. esculenta* peptides.

Peptide ID	Amino Acid Sequence	Observed (*m*/*z*)	Chargenumber	MW (Da)	Purity
SeP1	DEIHDLTDQLGEGGR	575.95	2	1654.72	95.9%
SeP2	DVEDLEAGLAK	580.30	2	1159.27	98.2%
SeP3	SPAFPELIEK	622.30	2	1130.32	98.4%
SeP4	SILAPNAIPGGFADGK	764.40	2	1527.75	97.6%
SeP5	EITSLAPSTM	525.26	2	1049.22	99.0%
SeP6	AALEEAEAALE	558.77	3	1116.20	98.2%
SeP7	NLNADIDGIR	550.78	2	1100.21	97.6%
SeP8	MQELVDKLQNK	449.24	2	1345.59	99.0%
